# The Development of Critical Care Medicine in China: From SARS to COVID-19 Pandemic

**DOI:** 10.1155/2020/3956732

**Published:** 2020-08-11

**Authors:** Haiyan Yin, Shan Wang, Youfeng Zhu, Rui Zhang, Xiaoling Ye, Jianrui Wei, Peter C. Hou

**Affiliations:** ^1^Department of Intensive Care Unit, The First Affiliated Hospital of Jinan University, Guangzhou 510000, Guangdong, China; ^2^Department of Intensive Care Unit, The Seventh Affiliated Hospital, Sun Yat-sen University, Shenzhen 518107, Guangdong, China; ^3^Department of Intensive Care Unit, Guangzhou Red Cross Hospital, Medical College, Jinan University, Guangzhou 510220, Guangdong, China; ^4^Guangzhou Women and Children's Medical Center, Guangzhou 510623, Guangdong, China; ^5^Division of Emergency Critical Care Medicine, Department of Emergency Medicine, Brigham and Women's Hospital, Boston, MA, USA; ^6^Harvard Medical School, Boston, MA, USA

## Abstract

**Background:**

Critical care medicine is a branch of medical science that deals with the characteristics and regularity of life-threatening processes initiated by any injury or disease and, accordingly, relevant treatment for patients with critical illness. Conceptions of critical care medicine in China stemmed in the early 1970s. Ever since the establishment of the first intensive care unit (ICU) along with the increasingly incomparable role of ICU in medical practices, critical care medicine has become an indispensable part of the Chinese medical and health system. Currently, critical care medicine as a secondary clinical discipline and a well-constructed science is in sustainable development on the way towards systematization and standardization.

**Methods:**

The gross domestic product (GDP) and population data were obtained from the National Bureau of Statistics. The number of ICUs, ICU beds, and hospital beds and other data regarding ICU staffing and facility resources were obtained from the Yearbook of Health in the People's Republic of China and National Bureau of Statistics. The mortality rates of SARS and COVID-19 and the number of health workers aiding Hubei amid COVID-19 pandemic were obtained from the National Health Commission. *Findings*. Critical care medicine in mainland China has made significant strides: both quantity and quality are progressing at a fast pace after SARS in 2003. Although there exist some disparities in healthcare personnel and medical resources, they have not hindered the country from mobilizing its healthcare workers and resources against a public health emergency.

## 1. Introduction

The first ICU in mainland China was set up in the Peking Union Medical College Hospital in 1982 [1]. Over the past decade, China's medical care development has made great strides. In 2005, the Chinese Society of Critical Care Medicine (CMA-CSCCM) was founded in Beijing [2], which was a milestone in the development of critical care medicine in China. Soon after, the first national ICU construction standard, “Guideline for the establishment and management of intensive care unit, China,” was issued by the CMA-CSCCM in 2006 [3].

**Figure 1:**
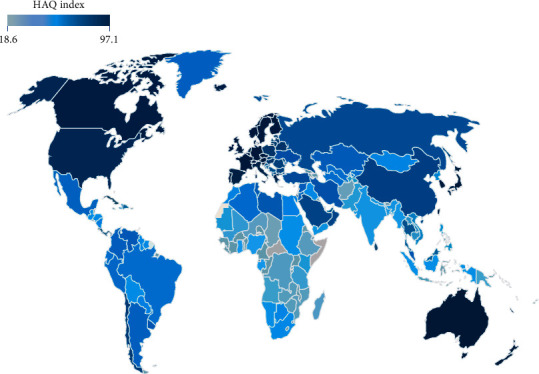
Global HAQ index in 2016 [5]. HAQ (healthcare access and quality) index: the index of each country is distributed between 0 and 100. The higher the score, the higher the chance and quality of medical services received by individuals in the country.

China's critical care medicine developed rapidly during the SARS epidemic. From 2003 to 2018, the medical investment and construction achievements have increased significantly, the per capita health expenditure has increased from ￥510 to ￥4,237, the number of health technicians per 1,000 population has increased from 3.33 to 6.83, and the number of beds in health institutions per 1,000 population has increased from 2.45 to 6.02 [4]. According to the data released by the *Lancet* on the Global Burden of Disease Study 2016 (Figure 1), China's healthcare access and quality (HAQ) index rose from 53.3 in 2000 to 77.9 in 2016, entering the first quartile of the countries in the world [5].

However, the uneven development of medical resources within regions of China remains a major problem. For example, in 2016, Beijing's HAQ index was at the top tenth of the countries in the world, while the western provinces and cities were mostly ranked at the bottom half of the world [5]. Besides that, shortage of nurses remains unsolved. According to the World Health Organization, the number of nurses in the world averaged at about 58 per 10,000 people in 2017: the highest was found in Norway (181), followed by the United States (145), the United Kingdom (82), and South Korea (71), while China had 26 [6], which was far below the global average.

Although these imbalances exist, it does not mean that the country is incapable of handling public health emergency. During this pandemic of COVID-19, China dispatched tens of thousands of healthcare workers from across the country to aid Hubei Province, the region hit hardest by COVID-19. Although the pandemic is still continuing, China has taken control of the spread by adhering to the principles of emergency preparedness: identify, inform, and isolate.

In this article, a summary of the variation and distribution of critical care resources in China from 1999 to 2018 is provided to describe how they were utilized against a public health emergency.

## 2. Methods

Existing data on critical care resources of public hospitals from 1999 to 2018 in the 31 province-level administrative units that are categorized under three regions in China were collected. The GDP and population data were obtained from the National Bureau of Statistics. The number of ICUs, ICU beds, and hospital beds and other data regarding ICU staffing and facility resources were obtained from the Yearbook of Health in the People's Republic of China and National Bureau of Statistics. The mortality rates of SARS and COVID-19 and the number of health workers aiding Hubei amid COVID-19 pandemic were obtained from the National Health Commission.

**Figure 2:**
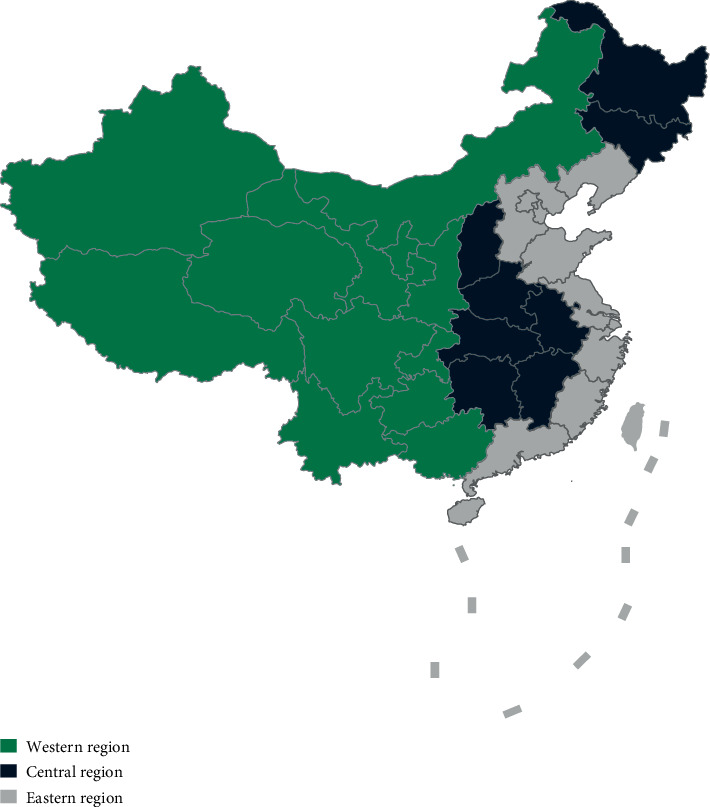
The western, central, and eastern regions of China.

The three regions divided into 31 province-level administrative units are as follows: the western region includes 12 units (Figure 2): Inner Mongolia, Chongqing, Guangxi, Sichuan, Guizhou, Yunnan, Tibet, Shaanxi, Gansu, Qinghai, Ningxia, and Xinjiang; the central region includes 8 units: Shanxi, Jilin, Heilongjiang, Anhui, Jiangxi, Henan, Hubei, and Hunan; and the eastern region includes 11 units: Beijing, Tianjin, Hebei, Liaoning, Shanghai, Jiangsu, Zhejiang, Fujian, Shandong, Guangdong, and Hainan.

## 3. Results

### 3.1. Number of Hospitals and ICUs

According to Table 1, in 2002, the number of hospitals among the three regions was not significantly different and ranged from 5,500 to 6,500. However, regional differences became significant in 2018; the number of hospitals in the east had nearly doubled in 2018, while the central region had fewer than 10,000 hospitals. The data about the number of ICU beds in each region were incomplete in 2002, but there was a remarkable rise in the total number, which was almost threefold in 2018. Furthermore, the total number of ICU beds in the three regions had no distinct difference in 2018 and reached an average of about 15,000 ICU beds. The number of ICU beds per 100,000 population also exhibited a positive linear correlation with the number of hospital beds per 100,000 population and GDP per capita in all regions.

### 3.2. Staffing to Care for Critically Ill Patients

In China, ICU doctors generally work 24 hours per shift, while nurses work 8 hours per shift, and each nurse is responsible for only one patient, so at least three nurses are needed for each bed per day. Based on these data, the Ministry of Health issued the “Guidelines for the Construction and Management of Critical Care Medicine in China,” which stated that the ratio of doctors tobeds should be greater than 0.8 : 1 and the ratio of nurses specializing in critical medicine to ICU beds should be 2.5–3 : 1 or more [3].

As shown in Table 1, the ratio of doctors to beds in all three regions was higher than 0.8 : 1 in 2018 and the ratio reached 2 : 1 in the eastern region. In contrast, the nurse-to-bed ratios in all three regions were lower than 3 : 1 in 2002 and 2018, indicating that the shortage of ICU nurses remained unsolved.

### 3.3. Advanced Education of Specialists in Critical Care Medicine

The disparity also exists in the education background of ICU staff among the three regions. The training of specialists in critical care medicine in China began in 2010, which required residents to receive a 3-year standardized training after graduating from the medical school, and at the end of the 3rd year of training, the examinations on theoretical and practical knowledge were required. After that, the residents need to take the Chinese Critical Care Certificate Course (5C) held by Chinese Society of Critical Care Medicine (CMA-CSCCM) to become an intensivist. Now, more than 20,000 ICU doctors in China have obtained the 5C certification [8]. In general, ICU staff in the more developed areas like the eastern region with more financial support have better education background and training opportunities compared with those in the west.

### 3.4. Facility Resources and Technological Equipment

The general investigation in critical care medicine is conducted every 5 years since 2005, so the nationwide statistics of available facility resources and technological equipment in 1999 and 2015 were compared. As shown in Table 2, in 2015, almost every ICU was equipped with noninvasive ventilators and blood gas monitors. Although some advanced medical instruments such as bronchoscope, bedside X-ray machine, and ultrasound were not available in most of the ICUs in 1999, there was a sharp increase from 1999. As for extracorporeal membrane oxygenation (ECMO) and intra-aortic balloon pumps, the penetration was still low.

### 3.5. Principles of Infection Control and Management of SARS and COVID-19

#### 3.5.1. Containing the Source of Infection and Expanding Treatment Range

In Wuhan, the capital of Hubei Province and epicenter in China, two hospitals—Huoshenshan and Leishenshan—modeled after the Beijing Xiaotangshan SARS Hospital, each with a capacity of more than 1,000 beds, were constructed in 10 days. Through the construction of makeshift hospitals, the expansion of designated hospitals, and the conversion of general hospitals, more than 100,000 beds were added in a short time.

Patients with serious and mild symptoms were separated to contain the source of infection. Critically ill patients were treated in designated hospitals, and advanced instruments like ECMO systems and ventilators were supplied to 46 such designated hospitals across the country. At the same time, exhibition centers, gymnasiums, and other facilities in Wuhan were converted into makeshift hospitals for patients with mild symptoms. The 16 makeshift hospitals in Wuhan admitted up to 12,000 patients with mild symptoms, accounting for more than a quarter of the infected patients [11]. Through centralized isolation and treatment for patients with mild symptoms, the chances of cross infection were largely reduced.

#### 3.5.2. Allocation of Resources and Guarantee of Supplies

**Figure 3:**
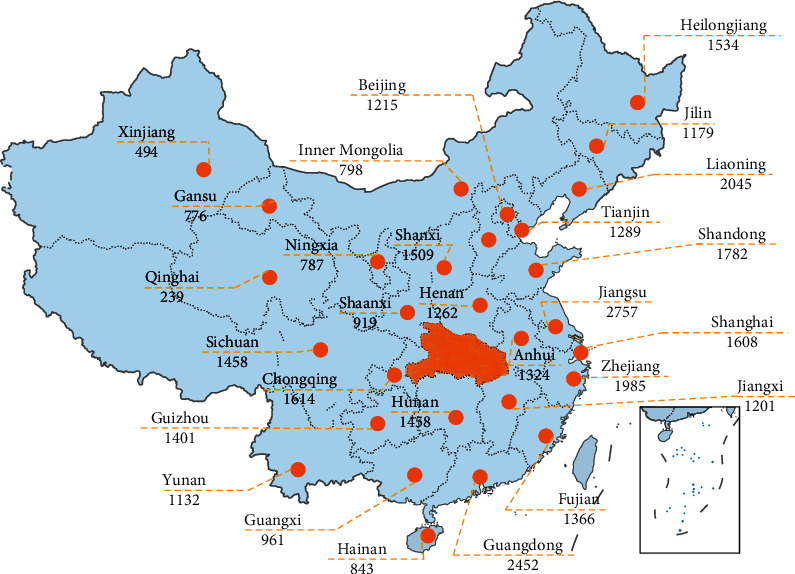
Healthcare workers dispatched to assist Hubei Province amid COVID-19 pandemic [13].

The mortality rate of COVID-19 before January 10 in mainland China was 17.3% in the early stages of the outbreak [12]. The experience gained from relief efforts during the SARS epidemic in 2003 and Wenchuan earthquake in 2008 was used this time, among which was “pairing assistance.” Professional healthcare workers from across the nation were dispatched to alleviate the stress and adversity caused by depletion of medical resources in severely affected areas. Since January 24, 2020, China has dispatched 42,600 healthcare workers grouped into 346 medical teams from across the country to Hubei Province (Figure 3). Over 35,000 healthcare workers were dispatched to assist Wuhan, the capital of Hubei Province and epicenter in China. Another 7,000 healthcare workers from 16 provincial-level regions offered partnership assistance to 16 other cities and prefectures in Hubei. Among the 42,600 healthcare workers sent to aid, about 19,000 (45%) had critical care training and expertise, including doctors and nurses, and 28,600 (67%) nurses were from different specialties. Over 12,000 (28%) were born after 1990, and 28,000 (65%) were female [13]. With the nationwide assistance, the mortality rate continued to decrease. On February 1, the mortality rate in mainland China dropped to 0.7% [12].

#### 3.5.3. Treatment Plan and Facility

Compared with SARS in 2003, the medical teams are more targeted and effective. Personalized treatment plan was prescribed after consultation with a multidisciplinary team consisting of experts on infections, respiratory illness, intensive care, cardiovascular diseases, and others. Furthermore, respiratory therapists and physiotherapists also participated in medical teams, playing an important role in patients' airway management and lung function rehabilitation. An array of standards was formulated for nursing critically ill patients, such as prone position mechanical ventilation, mechanical auxiliary expectoration, and fiberoptic bronchoscopy [11]. Many advanced instruments and technologies, including the invasive ventilator and noninvasive ventilator, high-flow nasal cannula, bedside hemofiltration, and ECMO, were put into use this time, allowing healthcare workers to treat critically ill patients efficiently.

### 3.6. Outcome and Mortality Rate of Critically Ill Patients

At the end of 2003, a total of 5,327 clinically diagnosed cases of SARS were reported in mainland China, of which 4,959 cases were cured and discharged, 349 died, and 19 cases died due to other diseases (not included in the number of SARS deaths) [14]. About 19 to 34% of SARS patients required admission to ICU, 13 to 26% required assisted ventilation, 20 to 22.6% developed ALI or ARDS, and 3.6 to 10.1% died at day 21 to day 28. The reported mortality in ICU was 34% at 28 days and 52.2% at 13 weeks [15].

As for COVID-19, about 80% of the laboratory-confirmed cases have prompt resolution of fever and pneumonitis with treatment and even without specific treatment in a minority. As of February 20, 2020, about 13.8% of COVID-19 patients developed ALI or ARDS and 6.1% of patients developed respiratory failure, septic shock, or MODS (multiple organ dysfunction syndrome) [12].

However, the regional disparity still exists in the mortality rate: areas with more developed medical resources have lower mortality rates. As shown in Table 3, the mortality rate of SARS in the eastern, western, and central regions of China was about 6.330%, 9.943%, and 6.130% separately, while the mortality rate of COVID-19 is about 0.743%, 0.944%, and 6.194%, with more than 80,000 diagnosed cases as of May 6 [16]. At the same time, the number of healthcare workers dispatched to assist Hubei Province from the eastern, western, and central regions is 18,432, 11,169, and 9,467, respectively [13]. Although about 47.2% of healthcare workers aiding Hubei Province come from the east, the mortality rate of it is the lowest, which is consistent with the uneven distribution of medical resources and ICU equipment availability among regions.

## 4. Discussion

As an overview of critical care resources in the western, central, and eastern regions of China, this report revealed a significant disparity among the three regions, not only in the distribution of ICU beds but also in the imbalance of ICU staff and resources.

The ratio of ICUs to hospital beds recommended by the Guidelines for the Construction and Management of Critical Care Medicine in China was 2–8%, while most of the surveyed hospitals did not meet this recommendation. The rapid expansion of hospital beds was disproportionate to the severe shortage of ICU beds. Moreover, the ratio of nurses to beds in ICUs was generally lower than the recommendation set by the guideline. In the western region, the ratio of doctors to beds was lower than 0.8 : 1. It is of great concern that heavy workload in the ICUs has resulted in staff fatigue and burnout, potentially affecting the care of critically ill patients [17]. Many studies on intensivists showed that long working hours, night shift, work conflict, and intensive environment were the main sources of psychological stress and burnout [18, 19].

At the same time, there is still a shortage of critical care technicians in China, such as respiratory therapists. The first respiratory therapy major in mainland China was founded in the Sichuan Huaxi Medical University in 1997 [20]. After many years of development, many medical schools have established the major of respiratory therapy, but currently the number of professional respiratory therapists in China is less than 1,000 [21]. Most of them work in the first-tier cities in the eastern region like Beijing, Shanghai, and Guangzhou. The lack of corresponding treatment and certification may be some of the biggest hindrances in the development of respiratory therapy. In order to promote the education, training, and number of specialists in critical care medicine, the government will need to increase the financial support in order to retain these ICU specialists in training.

Consistent with the uneven distribution of medical resources is ICU equipment availability among regions. The available facilities and equipment are relatively adequate in the eastern region, where there are continuous economic development and investment in the medical sector. However, some medical instruments such as bronchoscope, extracorporeal membrane oxygenation, and intra-aortic balloon pump were unavailable in some ICUs even in the developed region in 2015, which were barriers to rescuing critically ill patients. With the rapidly growing aging population in China, it is anticipated that the need for critical care resources will increase and the shortage of critical care resources will become more evident than what is observed now.

Just as the old Chinese adage states, one should “sharpen the tool before tackling a task,” the government should make every effort to enhance the scale of investment into critical care services and infrastructure to reduce the disparities in critical care education, training, and resources among the regions.

## 5. Conclusions

After the epidemic of SARS in 2003, critical care medicine in mainland China has made significant strides, and both quantity and quality are progressing at a fast pace. Although there exist some disparities in healthcare personnel and medical resources, they have not hindered the country from mobilizing its healthcare workers and resources against a public health emergency. When facing unexpected epidemic, the healthcare workforce caring for the patients, the operational efficiency of the government, the industrial capacity to produce medical supplies and hospitals, and the solidarity of the Chinese people are all factors to determine whether a country can effectively minimize the burden from an infectious epidemic.

## Figures and Tables

**Table 1:** Medical resources in the the western, eastern, and central regions of China in 2002 and 2018 [4, 7].

Medical resources	Western		Eastern		Central		Total	
	2002	2018	2002	2018	2002	2018	2002	2018
Hospitals	5,507	10,492	6,543	13,036	5,794	9,481	17,844	33,009
Hospital beds	768,757	1,869,563	1,218,946	2,626,720	919,450	2,023,466	2,907,153	6,519,749
ICU beds	—	13,536	—	17,510	—	14,822	1,934	45,868
ICU doctors	—	19,064	—	34,661	—	22,025	2,955	75,750
ICU nurses	—	19,731	—	31,692	—	21,076	3,625	72,499
Population (million)	366.91	379.56	482.32	581.09	425.96	435.88	1,275.19	1,396.53
GDP (billion)	2,008.09	18,431.89	6,828.91	50,631.80	2,965.07	22,409.42	11,802.07	91,471.41

**Table 2:** ICU equipment availability in China in 1999 and 2015 [9, 10].

ICU equipment	1999 (%)	2015 (%)
Bedside monitor	97.4	99.8
Invasive ventilator	84.8	96.7
Infusion pump	79.7	99.4
Noninvasive ventilator	80.0	98.6
Portable cardiac monitor	—	92.6
Portable ventilator	—	83.6
Defibrillator	83.3	99.4
Bronchoscope	22.4	65.9
Hemodynamic monitoring device	50.9	84.6
Bedside ultrasound	—	65.4
Extracorporeal membrane oxygenation	0.0	13.5
Intra-aortic balloon pump	9.2	17.4
Bedside X-ray machine	—	73.6
Blood gas monitor	35.2	97.3

**Table 3:** The number of confirmed cases and mortality rate of SARS and COVID-19 [14, 16].

Region		Confirmed cases		Mortality rate	
		SARS	COVID-19	SARS (%)	COVID-19 (%)
Eastern	Beijing	2,521	593	7.656	1.518
	Tianjin	175	190	8.000	1.579
	Hebei	215	328	5.581	1.829
	Liaoning	7	146	28.571	1.370
	Shanghai	8	656	25.000	1.067
	Jiangsu	7	653	0.000	0.000
	Zhejiang	4	1,268	25.000	0.079
	Fujian	3	356	0.000	0.281
	Shandong	1	788	0.000	0.888
	Guangdong	1,512	1,588	3.836	0.504
	Hainan	0	168	—	3.571
	Total	4,453	6,734	6.330	0.743

Western	Inner Mongolia	282	201	9.929	0.498
	Chongqing	3	579	0.000	1.036
	Guangxi	22	254	13.636	0.787
	Sichuan	20	561	10.000	0.535
	Guizhou	0	147	—	1.361
	Yunnan	0	185	—	1.081
	Tibet	0	1	—	0.000
	Shaanxi	12	306	0.000	0.980
	Gansu	8	139	12.500	1.439
	Qinghai	0	18	—	0.000
	Ningxia	5	75	20.000	0.000
	Xinjiang	0	76	—	3.947
	Total	352	2,542	9.943	0.944

Central	Shanxi	448	198	5.357	0.000
	Jilin	35	112	17.143	0.893
	Heilongjiang	0	944	—	1.377
	Anhui	10	991	0.000	0.605
	Jiangxi	1	937	0.000	0.107
	Henan	15	1,276	0.000	1.724
	Hunan	6	1,019	16.667	0.393
	Hubei	7	68,128	14.286	6.623
	Total	522	73,605	6.130	6.194
